# MicroRNA Expression and Carotid Plaque Vulnerability: An Exploratory Tissue-Based Study

**DOI:** 10.3390/jpm16050236

**Published:** 2026-04-28

**Authors:** Lucia Scurto, Ottavia Borghese, Giovanni Tinelli, Guido Rindi, Roberto Pola, Yamume Tshomba

**Affiliations:** 1 Unit of Vascular Surgery, Fondazione Policlinico Universitario A. Gemelli IRCCS, 00168 Rome, Italy; 2 Università Cattolica del Sacro Cuore, Largo Agostino Gemelli, 8, 00168 Rome, Italy; 3 Section of Anatomic Pathology, Department of Life Sciences and Public Health, Università Cattolica del Sacro Cuore, 00168 Rome, Italy; 4 Section of Internal Medicine and Thromboembolic Diseases, Department of Medicine and Geriatrics, Fondazione Policlinico Universitario A. Gemelli IRCCS, Università Cattolica del Sacro Cuore, 00168 Rome, Italy

**Keywords:** biomarkers, miRNA, carotid, atherosclerotic plaque, vulnerable plaque, stroke

## Abstract

**Background:** Reliable preoperative identification of carotid plaque instability remains challenging. Although duplex ultrasound allows early detection of carotid stenosis, it does not consistently predict plaque biological behavior. MicroRNAs (miRNAs) are small non-coding RNAs that regulate gene expression and have been implicated in atherosclerotic progression and plaque destabilization. The tissue-level expression of miRNAs in carotid plaques and their relationship with histological vulnerability remain incompletely defined. **Methods:** This exploratory, pilot, hypothesis-generating study included patients undergoing carotid endarterectomy for asymptomatic high-grade carotid stenosis (>75% NASCET). Plaque vulnerability was assessed using a multiparametric approach combining preoperative duplex ultrasound features (including Gray Scale Median, GSM), intraoperative macroscopic evaluation, and a validated histological scoring system; only plaques with concordant classification across all three modalities were retained for molecular analysis. Total RNA including small RNA was extracted from plaque tissue and miRNA expression was measured by qRT-PCR on a panel of 47 candidate miRNAs. Data were analyzed descriptively. **Results:** Twenty-eight patients were initially enrolled; after application of strict vulnerability criteria, five plaques (three unstable, two stable) were selected for miRNA profiling. Among the 47 miRNAs assayed, miR-122 and miR-197 showed a consistent descriptive trend toward higher expression in plaques classified as unstable; these plaques also displayed histological features of vulnerability (lipid-rich necrotic cores and inflammatory infiltrates). Given the extremely limited sample size, no inferential statistical comparisons or multiple-testing corrections were performed. **Conclusions:** In this small, tissue-based exploratory analysis, miR-122 and miR-197 were more highly expressed in plaques with histological features of instability. Due to the small sample size, the effect estimates are unstable, and the findings should be used solely to inform the design and power calculations of future studies. We outline the need of a clear, pragmatic validation pathway based on replication in independent, larger cohorts with standardized tissue handling and blinded assessment and parallel evaluation of circulating miRNA levels to assess noninvasive biomarker potential. Indeed, these findings are preliminary and strictly hypothesis-generating; validation in larger, prospectively collected cohorts and integration with circulating biomarkers and imaging data are required before clinical application.

## 1. Introduction

Atherosclerotic plaque instability represents the principal pathological substrate underlying acute cardiovascular and cerebrovascular events, including myocardial infarction and ischemic stroke [1,2].

Recent comprehensive reviews have synthesized mechanistic and clinical evidence linking plaque morphological features with downstream thromboembolic risk, underscoring the multifactorial nature of vulnerability that extends beyond luminal stenosis [3].

Although traditional cardiovascular risk factors and imaging modalities provide important prognostic information, they often fail to fully capture the complex biological mechanisms governing plaque progression and rupture [4,5,6]. Consequently, there is growing consensus that multimodal approaches combining imaging phenotypes with molecular signatures are required to improve individual risk prediction and to guide personalized therapeutic strategies.

Increasing evidence indicates that plaque vulnerability is not solely determined by structural features, such as fibrous cap thinning or lipid core enlargement, but rather by dynamic molecular and cellular processes, including inflammation, endothelial dysfunction, immune cell activation, neo-angiogenesis, and extracellular matrix remodeling [7,8,9,10]. These processes interact in spatially and temporally heterogeneous ways within the lesion microenvironment, producing distinct vulnerability endotypes that may respond differently to medical or interventional therapies [3].

In this context, microRNAs (miRNAs) have emerged as key post-transcriptional regulators of gene expression involved in cardiovascular homeostasis and disease. MiRNAs are small non-coding RNAs that modulate gene expression through mRNA degradation or translational repression [1]. Functionally, miRNAs act as nodal regulators that can coordinate multiple downstream pathways simultaneously, which makes them attractive both as mechanistic biomarkers and as potential therapeutic targets.

They are implicated in multiple molecular pathways relevant to atherosclerosis, including lipid metabolism, inflammatory signaling, immune system regulation, endothelial function, vascular smooth muscle cell behavior, and angiogenesis [2,3,4,5,6,7,8,9,10].

Experimental and translational studies have demonstrated that perturbation of specific miRNAs alters lesion composition and stability in preclinical models, providing proof-of-concept for miRNA-directed interventions [8].

Several miRNAs have been shown to regulate endothelial integrity, macrophage polarization, foam cell formation, and vascular smooth muscle cell proliferation, processes that collectively contribute to plaque growth and destabilization. Altered miRNA expression patterns have been associated with histological features of vulnerable plaques, such as inflammatory infiltrates, neovascularization, and increased proteolytic activity. These observations suggest that miRNA profiling may offer biologically relevant information beyond conventional morphological or imaging-based classifications. Moreover, the cell-type specificity of certain miRNAs permits more granular inference about the dominant pathogenic processes within a given plaque (for example, macrophage-driven inflammation versus smooth muscle cell-mediated fibrous cap remodeling) [8].

While genomic and transcriptomic studies have been extensively conducted in coronary artery disease, comparatively limited data are available for carotid atherosclerosis. Nonetheless, recent investigations have reported that specific miRNAs, including miR-100, miR-127, miR-133a, and miR-145, are overexpressed in symptomatic carotid plaques [11]. These human tissue-based observations have been corroborated by independent cohorts and are consistent with mechanistic data implicating these miRNAs in processes such as smooth muscle cell phenotype switching and extracellular matrix turnover [12].

In addition, molecular analyses of carotid endarterectomy specimens have demonstrated differential expression of miR-21 and miR-143 between symptomatic and asymptomatic lesions [13]. Notably, miR-21 has been reported to exert context-dependent effects on plaque biology, modulating both reparative and destabilizing pathways, which highlights the need for cell-specific and temporally resolved analyses when interpreting miRNA dysregulation [13].

Despite these promising findings, the translational applicability of tissue-based miRNA signatures for discriminating stable from unstable carotid plaques remains incompletely defined. At present, no molecular biomarker has been validated for routine clinical use, and preoperative imaging continues to represent the primary tool for plaque assessment. A deeper understanding of miRNA-mediated regulatory networks within carotid atherosclerotic lesions may therefore contribute to improved risk stratification and, potentially, to the identification of novel therapeutic targets. To achieve clinical translation, future studies must address reproducibility across centers, standardization of tissue handling and assay platforms, and the incremental prognostic value of miRNA signatures over established clinical and imaging predictors [8].

Within this framework, the aim of the present exploratory study was to investigate the expression of selected miRNAs in carotid atherosclerotic plaques and to examine their relationship with plaque vulnerability, as defined by a combined imaging and histopathological assessment. This preliminary work seeks to identify molecular patterns associated with plaque instability and to generate hypotheses for future validation studies.

Ultimately, integrating lesion-level molecular data with circulating miRNA profiles and advanced imaging phenotypes may enable the development of minimally invasive assays for dynamic monitoring of plaque behavior and for stratifying patients who might benefit from intensified medical therapy or earlier revascularization.

## 2. Materials and Methods

### 2.1. Study Design and Patient Selection

**Figure 1:**
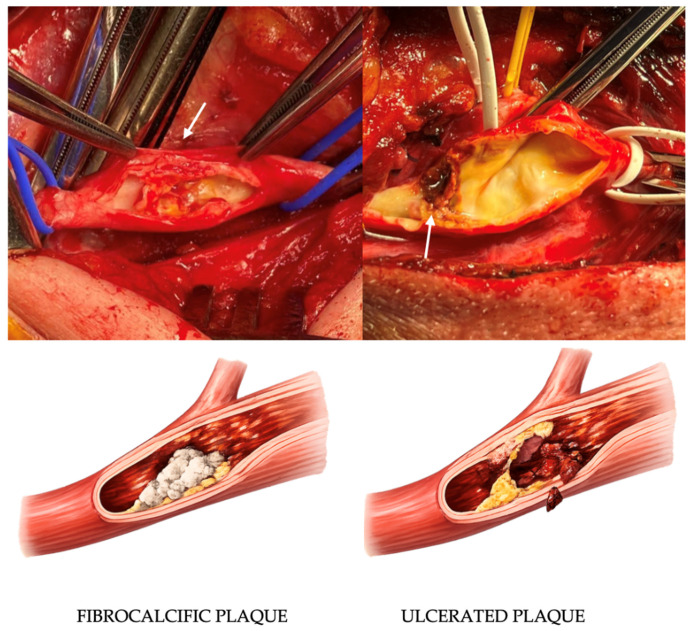
Intraoperative images and schematic representation of a fibrocalcific (on the (**left**)) or fibrolipidic ulcerated plaque (on the (**right**)) as indicated by the with arrow. A fibrocalcific, stable plaque displays a thick fibrous cap and dense calcifications, features typically associated with lower vulnerability and reduced risk of rupture. An ulcerated, lipid-rich, unstable plaque is conversely characterized by surface disruption, intraplaque hemorrhage, and irregular morphology consistent with high embolic potential.

This was an exploratory, pilot, hypothesis-generating study designed to investigate miRNA expression patterns in carotid atherosclerotic plaques with different degrees of vulnerability (Figure 1). This study was intentionally framed as a discovery-phase investigation to identify candidate molecular signatures rather than to provide definitive, generalizable effect estimates, and the analytical plan therefore emphasized internal validity and biological specificity over statistical power.

Consecutive patients undergoing carotid endarterectomy for asymptomatic carotid stenosis (>75% according to NASCET criteria on preoperative computed tomographic angiography) at the Vascular Surgery Unit of Agostino Gemelli Hospital between June and November 2019 were considered for inclusion. The threshold for surgical indication followed established trial-derived criteria and contemporary practice guidelines, ensuring that enrolled lesions represented clinically relevant high-grade stenoses.

All patients received a detailed explanation of the study and provided written informed consent prior to surgery. Baseline demographic characteristics and major comorbidities were recorded. To minimize inter-observer variability, all patients underwent a standardized preoperative duplex ultrasound examination performed at admission by the same experienced operator using the same ultrasound system. Preoperative ultrasound assessments included quantitative grayscale median (GSM) analysis and standardized plaque segmentation to provide an objective echogenicity metric that correlates with histological features of vulnerability.

The study was not designed to test predefined hypotheses or to provide definitive estimates of effect size. Rather, it aimed to generate preliminary biological insights through a highly controlled and restrictive analytical framework.

Accordingly, statistical analyses were exploratory and focused on effect directionality and biological plausibility; findings were interpreted as hypothesis-generating and intended to inform the design and sample-size calculations of subsequent confirmatory studies.

To minimize misclassification bias in this exploratory tissue-based analysis, we applied strict concordance criteria across three independent assessment modalities (preoperative duplex ultrasound including GSM, intraoperative macroscopic evaluation, and histological scoring). Only plaques with concordant classification as clearly stable or clearly unstable across all three modalities were retained for miRNA profiling.

This multimodal concordance approach mirrors contemporary recommendations for integrated plaque phenotyping and reduces the risk that molecular differences reflect sampling heterogeneity or modality-specific misclassification.

Cases were excluded if they displayed intermediate or discordant features between modalities or if tissue quality failed predefined RNA integrity.

This conservative approach prioritized biological specificity over sample size to increase the likelihood that observed molecular differences reflect true tissue-level differences between extreme phenotypes rather than classification noise.

### 2.2. Multiparametric Assessment of Plaque Vulnerability

Plaque vulnerability was assessed using a multiparametric approach combining preoperative imaging, intraoperative macroscopic evaluation, and histological analysis (Table 1).

Preoperative duplex ultrasound was performed using an iU22 scanner (Philips, Andover, MA, USA) equipped with an L12–5 MHz linear transducer. Each plaque was initially classified according to its predominant macroscopic composition as fibrotic, fibro-lipidic, fibro-calcific, or calcific (Table 2). These findings were subsequently correlated with intraoperative macroscopic observations.

**Figure 2:**
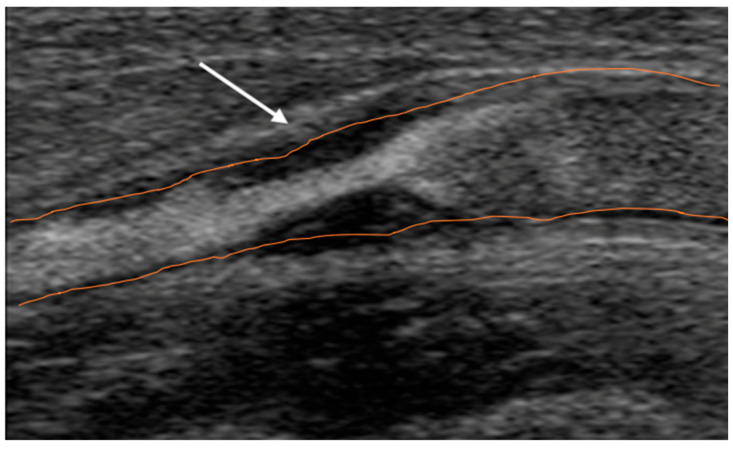
B-mode Doppler ultrasound image of carotid stenosis (arrow).

B-mode images were post-processed to calculate Gray Scale Median (GSM) [14]; a GSM cut-off of 25 was used to identify hypoechoic plaques, but GSM alone was not considered definitive. Intraoperative macroscopic evaluation and histological analysis were used in combination; discordant or intermediate cases were excluded from molecular analyses. Histology served as the tissue reference standard (Figure 2).

Following surgical excision, plaques were immediately fixed and frozen. Cryostat sections (20 μm thickness) were prepared, post-fixed, and stained with hematoxylin and eosin using standardized protocols. Histological evaluation was independently performed by two blinded observers using a validated multiparametric score incorporating 11 features of plaque instability [15]. These features include: (1) large lipid/necrotic core; (2) thin or ruptured fibrous cap; (3) intraplaque hemorrhage; (4) dense inflammatory infiltrate (macrophages/lymphocytes); (5) neovascularization; (6) presence of ulceration or surface rupture; (7) calcification pattern (spotty vs. dense); (8) cholesterol clefts; (9) increased proteolytic activity (morphological surrogates); (10) presence of foam cells; and (11) adventitial inflammatory reaction (Supplementary Table S1).

**Figure 3:**
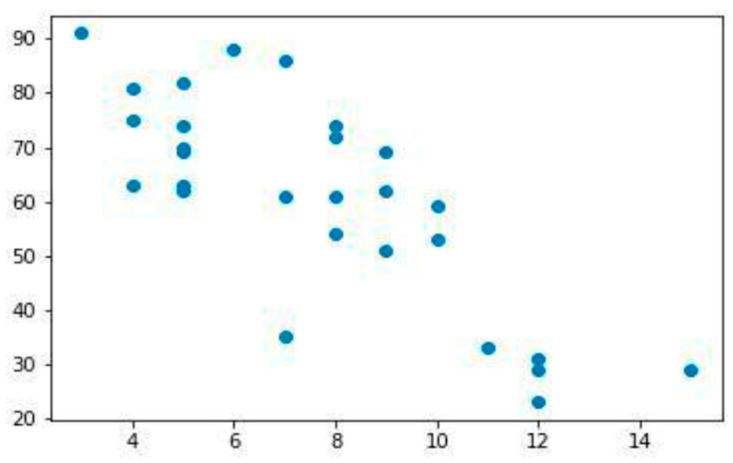
The expression of specific microRNAs in the surgical specimen was matched through a multivariate analysis to the stability scored of the corresponding plaque. To limit risk of bias, plaques identified as not certainly stable or not certainly unstable were excluded. In samples identified as certainly stable or certainly unstable, the expression of specific microRNAs in the surgical specimen was matched.

To reduce misclassification bias and enhance biological contrast, only plaques that could be confidently classified as clearly stable or clearly unstable based on concordant imaging and histological findings were included in downstream molecular analyses. Plaques with intermediate or ambiguous features were intentionally excluded (Figure 3).

### 2.3. miRNA Extraction and Expression Analysis

After excision, tissue specimens were stabilized immediately in RNAlater (Thermo Fisher Scientific, Waltham, MA, USA) and stored at −80 °C according to standardized biobanking procedures to preserve RNA integrity. Total RNA, including the small RNA fraction, was extracted from ~20–30 mg of tissue using the miRNeasy Mini Kit (Qiagen, Venlo, The Netherlands cat. 217004) following the manufacturer’s protocol with on-column DNase digestion. Homogenization was performed using a rotor-stator homogenizer in QIAzol Lysis.

RNA concentration and purity were measured by NanoDrop spectrophotometry (Thermo Fisher Scientific). RNA integrity and the small RNA fraction were assessed using the Agilent 2100 Bioanalyzer (Agilent Technologies, Inc., Santa Clara, CA, USA) with the RNA 6000 Nano and Small RNA kits; samples with RNA integrity number (RIN) < 5 or with evidence of extensive degradation were excluded from downstream analysis.

Candidate miRNAs (*n* = 47) were selected a priori based on prior experimental and clinical evidence linking them to pathways relevant to atherosclerosis (inflammation, lipid metabolism, endothelial function, angiogenesis, extracellular matrix remodeling). A complete list of the 47 miRNAs with a one-line rationale and key supporting references is provided in Supplementary Table S2.

Quantitative real-time PCR (qRT-PCR) was performed using array-based platforms using TaqMan Advanced miRNA Assays (Thermo Fisher Scientific) on a QuantStudio 7 Flex Real-Time PCR System (Applied Biosystems, Thermo Fisher Scientific, Foster City, CA, USA) with standard cycling conditions. qRT-PCR data were normalized to the geometric mean of two endogenous small RNA controls: U6 snRNA and RNU48. Reference gene stability was assessed using geNorm and NormFinder.

Relative miRNA expression levels were calculated using the comparative Ct (ΔΔCt) method and expressed as fold change relative to plaques classified as stable. Raw amplification curves were inspected and technical replicates with Ct differences >0.5 were re-assayed; persistent outliers were excluded. Assays with no amplification or Ct > 40 were considered undetermined.

Quality control criteria were predefined: samples showing aberrant amplification curves, poor reference gene stability, or excessive technical variability across replicates were excluded from analysis.

### 2.4. Statistical Analysis

Given the exploratory and preliminary nature of the study, statistical analyses were primarily descriptive and aimed at identifying consistent expression patterns with biological plausibility rather than testing formal hypotheses.

We attempted non-parametric comparisons (Mann–Whitney U test) and false discovery rate (FDR) adjustment; however, only five plaques (three unstable, two stable) met the strict inclusion criteria, rendering inferential testing and multiple-comparison correction statistically unreliable and potentially misleading. Therefore, no inferential *p*-values are reported for miRNA comparisons; fold changes are presented descriptively. No a priori sample size calculation was performed, as the study was not designed to estimate effect sizes or to support inferential conclusions.

Continuous variables are reported as mean ± standard deviation or median with interquartile range, as appropriate. Categorical variables are presented as absolute counts and percentages. Assessment of data distribution was performed using visual inspection and Shapiro–Wilk testing, acknowledging the limited reliability of normality testing in very small samples.

Due to the extremely limited number of plaques meeting strict vulnerability criteria, no inferential statistical comparisons between stable and unstable groups were performed, and no correction for multiple comparisons was applied. Fold changes in miRNA expression were interpreted descriptively and in relation to concordant histopathological features of plaque vulnerability.

This analytical strategy was intentionally adopted to minimize the risk of both type I and type II errors and to prioritize biological relevance over statistical significance. The results should be interpreted as hypothesis-generating and intended to inform the design and power calculations of future confirmatory studies.

## 3. Results

Twenty-eight patients (10 women, 35.7%; 18 men, 64.3%) with a mean age of 73.6 years were initially included. Dyslipidemia was present in all patients (100%), followed by hypertension (75%), ischemic heart disease (32%), diabetes mellitus (28.5%), and chronic obstructive pulmonary disease (25%) (Table 3).

After application of the strict multiparametric vulnerability criteria and quality-control thresholds, five plaques were classified with high confidence and selected for miRNA profiling: three as clearly unstable and two as clearly stable.

Several specimens were excluded because they displayed discordant features across imaging, macroscopic assessment, and histology, or because tissue RNA quality failed predefined thresholds.

Among the 47 miRNAs analyzed, miR-122 and miR-197 showed a consistent descriptive trend toward higher expression in plaques classified as unstable compared with stable plaques. Plaques exhibiting increased expression of these miRNAs were characterized histologically by a greater representation of lipid-rich necrotic cores and inflammatory cellular components.

Overall, given the limited sample size, these observations are presented descriptively and should be interpreted with caution. Indeed, due to the small sample size the effect estimates are unstable, and the findings should be used solely to inform the design and power calculations of future studies (Table 4).

## 4. Discussion

The widespread use of duplex ultrasound has facilitated early detection of carotid stenosis; however, reliable preoperative criteria for identifying plaque instability remain lacking. Recent systematic appraisals of the literature emphasize that conventional sonographic metrics capture structural and hemodynamic features but do not fully reflect the molecular and cellular processes that determine embolic potential. Lesions that are not hemodynamically severe may nonetheless carry a substantial embolic risk, potentially leading to severe neurological events [16]. This clinical paradox has driven interest in adjunctive biomarkers and integrated phenotyping strategies that can identify biologically high-risk plaques despite modest luminal narrowing. Consequently, there is an unmet need for complementary biological markers capable of refining plaque risk stratification. Such markers should ideally be mechanistically informative, reproducible across centers, and additive to established clinical and imaging predictors to justify translation into preoperative decision-making.

MicroRNAs are increasingly recognized as regulators of inflammatory signaling, lipid metabolism and vascular cell phenotypes relevant to atherogenesis and plaque destabilization. Their stability in tissue and biofluids, combined with the capacity to modulate multiple downstream targets, renders miRNAs particularly attractive as candidate biomarkers and as potential therapeutic modulators of plaque biology.

In this exploratory study we combined imaging, macroscopic and histological assessments with tissue-level miRNA profiling to reduce misclassification and to link molecular signatures to local histopathology. By anchoring molecular measurements to lesion-level histopathology, the study design aimed to increase biological specificity and to facilitate mechanistic interpretation of differential miRNA expression. While the GSM reflects plaque composition, its variability and limited prognostic specificity restrict its use as a standalone marker of vulnerability [17]. Moreover, imaging features alone cannot reliably predict plaque biological behavior over time. Our integrative framework acknowledges that no single modality is sufficient to define vulnerability and that concordant signals across modalities strengthen the inference that observed molecular differences are pathophysiologically meaningful.

Previous studies examining circulating miRNAs in symptomatic and asymptomatic patients have provided important insights [4,13], but are subject to potential confounding from systemic inflammatory conditions and the inclusion of clinically silent vulnerable plaques in control groups. Circulating miRNA signatures may therefore lack lesion specificity and can be influenced by extracerebral sources, comorbidities, and acute systemic responses. To address these limitations, the present study focused on direct analysis of plaque tissue, thereby linking miRNA expression to local histopathological features. Tissue-based profiling permits attribution of miRNA signals to the lesion microenvironment and enables correlation with cell-type-specific histological hallmarks such as macrophage infiltration, intraplaque hemorrhage, and neovessel density.

Among the 47 candidate miRNAs assayed, miR-122 and miR-197 emerged as candidate markers that were more highly expressed in plaques with histological features of vulnerability. The identification of these candidates in tissue supports their potential relevance to local pathogenic processes rather than reflecting systemic noise. This observation is biologically plausible: miR-122 has been implicated in lipid metabolism, angiogenesis and inflammatory signaling [18,19,20,21,22,23,24], while miR-197 has been associated with endothelial inflammation and angiogenic activation in experimental models [25]. The functional roles attributed to these miRNAs in preclinical studies—ranging from modulation of cholesterol handling to regulation of endothelial activation—provide mechanistic plausibility for their association with intraplaque neovascularization and proteolytic remodeling. Recent data further support the potential of miRNAs as molecular biomarkers of vascular inflammation and tissue instability [8,26,27]. Collectively, these lines of evidence justify prioritizing miR-122 and miR-197 for targeted mechanistic experiments and for evaluation in larger, prospectively collected human cohorts.

However, the present data are descriptive and hypothesis-generating. The exploratory nature of the analysis necessitates cautious interpretation and precludes claims about diagnostic accuracy or causal inference. The very small number of plaques analyzed precludes inferential conclusions about the reproducibility, effect size, or diagnostic performance of these miRNAs. Mechanistic interpretations linking miR-122 and miR-197 to plaque destabilization are speculative and should be tested in targeted experimental models and validated in larger human cohorts [25]. Robust validation will require standardized tissue processing, blinded assessment, and multicenter replication to assess inter-laboratory reproducibility and to quantify incremental prognostic value over existing clinical and imaging models.

More specifically, a clear, pragmatic validation pathway is required. We recommend replication in independent, larger cohorts with standardized tissue handling and blinded assessment; parallel evaluation of circulating miRNA levels to assess noninvasive biomarker potential; multicenter prospective registries to capture sufficient event rates and heterogeneity; and mechanistic follow-up in targeted experimental models to test causality. These are the necessary prerequisites before any translational or clinical claims can be made.

Future work should also determine whether tissue signals are reflected in circulating miRNA levels (plasma, microvesicles) and whether combined multimodal classifiers (imaging + circulating biomarkers + tissue profiling) improve preoperative risk stratification. If concordant tissue and circulating signatures are identified, they could enable minimally invasive monitoring and dynamic risk assessment, which are essential for clinical translation.

These mechanistic links, while preliminary, align with the histopathological correlates observed in our vulnerable plaque specimens and provide a focused rationale for subsequent mechanistic interrogation.

We emphasize once more that the small sample size of the present study limits any generalization and that our tissue observations are descriptive and hypothesis-generating; mechanistic roles of miR-122 and miR-197 in plaque destabilization should be tested in targeted experimental models and validated in larger human cohorts before translational claims can be made.

The present findings should not be interpreted as evidence of diagnostic or prognostic utility. Rather, they support the biological relevance of these miRNAs within the plaque microenvironment and provide a rationale for further investigation.

## 5. Limitations

The principal limitation of this study is the small number of plaques included in the molecular analysis, a consequence of the deliberate application of strict concordance criteria across imaging, macroscopic and histological assessments. While this approach reduces misclassification and increases biological contrast for discovery, it limits statistical power, reproducibility and generalizability. The small number of specimens constrains our ability to generalize findings beyond this cohort and increases the likelihood that observed patterns reflect idiosyncratic features of these samples rather than reproducible biological signals.

The extremely limited sample size precludes robust inferential statistics. We therefore present descriptive and exploratory analyses only and avoid formal hypothesis testing. Any apparent associations should be interpreted as preliminary observations that require confirmation in larger datasets.

We did not perform cell-type-specific localization of miRNA expression (e.g., laser microdissection or in situ hybridization), so the cellular source of the observed miRNA signal remains uncertain.

Comprehensive systemic biomarker data (e.g., Lp[a], lipoprotein subfractions, high-sensitivity CRP) were not uniformly available and therefore were not analyzed; this limits assessment of the relationship between local miRNA dysregulation and systemic lipid or inflammatory status.

Finally, the cross-sectional design prevents assessment of temporal changes in miRNA expression or direct correlation with clinical outcomes.

Future studies should increase sample size, include prospectively collected specimens, and apply appropriate statistical frameworks to assess reproducibility, effect sizes, and clinical relevance. Where feasible, orthogonal validation (e.g., independent assays or cohorts) should be used to confirm candidate findings.

These limitations underscore the hypothesis-generating nature of the findings and the need for larger, prospective, and multimodal validation studies.

## 6. Future Directions

Future studies should prospectively collect comprehensive lipid profiles (including Lp[a] and lipoprotein subfractions) and inflammatory biomarkers (e.g., hs-CRP) to evaluate correlations with tissue and circulating miRNA expression and to determine whether combined molecular and systemic biomarker panels improve risk stratification.

## 7. Conclusions

This exploratory tissue-based study identified miR-122 and miR-197 as candidate miRNAs more highly expressed in carotid plaques with histological features of instability. Given the very small sample size and the deliberate selection of concordant extreme phenotypes, these results are preliminary and strictly hypothesis-generating.

The findings should be used solely to inform the design and power calculations of future studies. We outline the need of a clear, pragmatic validation pathway based on replication in independent, larger cohorts with standardized tissue handling and blinded assessment and parallel evaluation of circulating miRNA levels to assess noninvasive biomarker potential.

Substantial validation in larger, well-characterized and prospective cohorts is required to determine reproducibility, clinical relevance, and potential utility as adjunctive biomarkers for preoperative risk stratification.

## Figures and Tables

**Table 1:** Multiparametric histological score used to evaluate plaque instability.

Histological Feature	Grade 0	Grade 1	Grade 2	Grade 3
Hemorrhage	No hemorrhage	Small hemorrhage	Large hemorrhage	-
Thrombus	No thrombus	Small Thrombus	Large Thrombus	-
Lipid Core	No lipid core	Small lipid core	Large lipid core	-
Fibrous Tissue	Very little fibrous tissue	Approx. 50%	Predominantly	-
Chronic Plaque Inflammation	None	Occasional cells ore one group ≥ 50	2–5 groups ≥ 50	≥5 groups ≥ 50 or 1 group ≥ 500
Chronic Cap Inflammation	None	<10 cells in cap	10–50 cells in cap	≥50 cells in cap
Acute Plaque Inflammation	None	Occasional cells ore one group ≥ 50	2–5 groups ≥ 50	≥5 groups ≥ 50 or 1 group ≥ 500
Acute Cap Inflammation	None	<10 cells in cap	10–50 cells in cap	≥50 cells in cap
Foam Cells	None	<50 cells	≥50 cells	-
Neovascularity	None	<10/section	≥10/section	-
Cap Rupture	Intact	Probably intact	Probably ruptured	Definitely ruptured

**Table 2:** Macroscopic aspect of the plaque and mean GSM in the population under analysis.

Macroscopic Aspect of the Plaque in Relation to GSM	N of Patients (Total 28)	%	Mean GSM Score
Ulcerated	2	7.1	45
Fibrolipidic plaque	5	17.8	56.8
Fibrocalcific plaque	18	64.2	63.2
Calcific plaque	3	10.7	62.6

**Table 3:** Baseline data.

	N of Patients (Total 28)	%
Demographics		
Age (years)	73.6 years	range 56–86 years
Gender	Female (10)	35.7
	Male (18)	64.3
Hypertension	21	75
Diabetes mellitus	9	28.5
CAD ^a^	9	32
Dyslipidaemia	28	100
Smoking habits	15	53.5
COPD ^b^	7	25
Preoperative drug		
Statin	27	96.4
Antiplatelet	28	100
Antihypertensive	21	75
Carotid Stenosis (NASCET ^c^ criteria)		
Ipsilateral Carotid stenosis 70–79%	16	57.1
Ipsilateral Carotid stenosis 80–85%	10	35.7
Ipsilateral Carotid stenosis 86–99%	2	7.1

^a^ CAD coronary artery disease. ^b^ COPD Chronic Obstructive Pulmonary Disease. ^c^ NASCET criteria: from the North American symptomatic carotid endarterectomy trial.

**Table 4:** Recommendations for designing future tissue-based and translational studies of microRNAs in carotid atherosclerotic plaques.

Recommendation	Rationale (from Current Study)	Concrete Implementation
Use a multiparametric plaque classification (imaging + macroscopic + histology)	The study used concordant GSM, intraoperative macroscopic evaluation and validated histological scoring to reduce misclassification.	Standardize ultrasound protocol (same transducer, GSM post-processing software), blinded macroscopic assessment, and dual independent histopathology reads. Record discordance rates.
Increase sample size and use prospective, multicenter cohorts	Small, single-center exploratory sample (5 profiled plaques) limited power and generalizability.	Perform sample-size calculation based on pilot fold-changes; recruit multicenter cohorts with predefined enrollment targets for stable vs. unstable plaques.
Preselect candidate miRNA panels and plan orthogonal validation	We assayed 47 candidate miRNAs and highlighted miR-122 and miR-197 as candidates; recommend orthogonal validation.	Predefine candidate list and validation criteria Stage 1: discovery panel (broad qPCR/RNA-seq). Stage 2: targeted validation (independent cohort) using qRT-PCR and an independent assay (e.g., digital PCR).
Integrate circulating miRNA measurements and systemic biomarkers	The paper recommends parallel evaluation of circulating miRNAs to assess noninvasive biomarker potential.	Collect matched plasma/serum (and microvesicle fractions) at time of surgery; measure same miRNAs in tissue and circulation; collect hs-CRP, Lp(a), lipoprotein subfractions.
Combine imaging, molecular and clinical endpoints in prospective registries	Authors propose multimodal classifiers (imaging + circulating + tissue) and multicenter registries to capture events.	Embed molecular substudies within prospective carotid registries capturing clinical outcomes (stroke, TIA, perioperative events) and serial imaging.

## Data Availability

The raw data supporting the conclusions of this article will be made available by the corresponding author on request.

## Supplementary Materials

The following supporting information can be downloaded at: https://www.mdpi.com/article/10.3390/jpm16050236/s1, Supplementary Table S1: Per sample multiparametric histology scores (11 features); Supplementary Table S2: Candidate miRNAs selected a priori (*n* = 47) [28,29,30,31,32].
