# Supplementary material for: MicroRNA Expression and Carotid Plaque Vulnerability: An Exploratory Tissue-Based Study
Source: J Pers Med. 2026 Apr 28;16(5):236. doi: 10.3390/jpm16050236 (PMC13208643; doi:10.3390/jpm16050236)
Supplement: Supplementary file 1 [file jpm-16-00236-s001.zip › jpm-4188738-supplementary.pdf]

Journal of
Personalized Medicine

Supplementary Table S1. Per-sample multiparametric histology scores (11 features)

Sample ID	Group	Lipid/necrotic core (0–3)	Fibrous cap (0–3)	Intraplaque hemorrhage (0/1)	Inflammatory infiltrate (0–3)	Neovascularization (0–3)	Ulceration/surface rupture (0/1)	Calcification pattern (spotty=1/dense=0)	Cholesterol clefts (0/1)	Proteolytic activity (0–3)	Foam cells (0–3)	Adventitial inflammation (0–3)	Total instability score
S01	Unstable	3	2	1	3	2	1	1	1	2	3	2	20
S02	Unstable	3	1	1	3	3	1	1	1	3	3	2	21
S03	Unstable	2	2	0	2	2	0	1	1	2	2	1	15
S04	Stable	1	3	0	1	0	0	0	0	1	1	0	7
S05	Stable	1	3	0	1	0	0	0	0	1	1	0	7

Supplementary Table S2 — Candidate miRNAs selected a priori (n = 47)

	miRNA	Rationale
1	miR-21	Promotes VSMC proliferation, inflammation and plaque progression.
2	miR-126	Endothelial-enriched; preserves endothelial integrity and angiogenic signalling.
3	miR-155	Central regulator of vascular inflammation and macrophage activation.
4	miR-146a	Negative regulator of NF-κB signalling and vascular inflammation.
5	miR-33a	Controls cholesterol efflux and HDL biogenesis via ABCA1/ABCG1 repression.
6	miR-33b	Paralog of miR-33a with similar effects on lipid metabolism.
7	miR-92a	Inhibits endothelial pro-angiogenic programmes and promotes dysfunction.
8	miR-92b	Modulates endothelial responses and inflammatory signalling.
9	miR-145	Drives VSMC contractile phenotype and plaque stability.
10	miR-143	Works with miR-145 to regulate VSMC differentiation and migration.
11	miR-223	Regulates myeloid cell function and inflammasome-related pathways.
12	miR-34a	Promotes endothelial senescence and VSMC phenotypic switching.
13	miR-125a-5p	Modulates macrophage lipid uptake and inflammatory cytokine production.
14	miR-125b	Influences VSMC proliferation and inflammatory gene expression.
15	miR-30c	Regulates hepatic lipid metabolism and circulating lipoprotein levels.
16	miR-27a	Controls lipid handling and inflammatory mediators in vascular cells.
17	miR-27b	Modulates angiogenesis and lipid metabolism in vascular contexts.
18	miR-10a	Anti-inflammatory in endothelium; suppresses pro-atherogenic gene programmes.
19	miR-181b	Inhibits endothelial NF-κB signalling and vascular inflammation.
20	miR-210	Hypoxia-responsive; affects angiogenesis and plaque neovascularization.
21	miR-221	Regulates endothelial proliferation and VSMC migration.
22	miR-222	Partner of miR-221; modulates endothelial and VSMC behaviour.
23	miR-378	Influences lipid metabolism and macrophage foam-cell formation.

 https://doi.org/10.3390/jpm16050236

24	miR-29a	Controls extracellular matrix remodelling via collagen and MMP regulation.
25	miR-24	Modulates endothelial apoptosis and inflammatory responses.
26	miR-17-5p	Part of miR-17~92 cluster; affects endothelial proliferation and inflammation.
27	miR-19b	Regulates inflammatory signalling and VSMC phenotype within the cluster.
28	miR-320	Linked to endothelial dysfunction and metabolic regulation in vascular cells.
29	miR-146b	Similar to miR-146a; dampens inflammatory signalling in vascular cells.
30	miR-195	Implicated in VSMC proliferation and extracellular matrix changes.
31	miR-199a	Modulates hypoxia responses and VSMC behaviour.
32	miR-214	Regulates angiogenesis, VSMC survival and inflammatory pathways.
33	miR-342-3p	Influences lipid efflux and foam-cell formation in macrophages.
34	miR-150	Affects macrophage polarization and cholesterol handling.
35	miR-133a	Modulates endothelial NO signalling and VSMC phenotype.
36	miR-615-5p	Reported regulator of eNOS activity and endothelial function.
37	miR-138	Influences eNOS and endothelial nitric oxide bioavailability.
38	miR-370	Linked to lipid metabolism and atherogenic lipoprotein regulation.
39	miR-486	Associated with vascular inflammation and endothelial responses.
40	miR-10b	Modulates endothelial inflammation and VSMC migration.
41	miR-30a	Regulates VSMC apoptosis and extracellular matrix remodelling.
42	miR-451	Impacts macrophage activation and oxidative stress responses.
43	miR-16	Broad regulator of cell cycle and apoptosis in vascular cells.
44	miR-29b	ECM remodelling and collagen regulation relevant to plaque stability.
45	miR-29c	Member of miR-29 family; affects fibrosis and matrix turnover.
46	miR-197	Associated with immune cell activation and plaque inflammation
47	miR-122	Linked to lipid metabolism and inflammatory signalling in vascular tissue

Key supporting references used to curate the 47-miRNA list

- ***Li Z***, *Zhao Y, Suguro S, Suguro R. MicroRNAs Regulate Function in Atherosclerosis and Clinical Implications. Oxid Med Cell Longev. 2023;2023:2561509. doi:10.1155/2023/2561509.*
- ***Silva VR***, *Azar A, Goncalves ER, de Moura Nascimento TC, Buchaim RL, Buchaim DV, et al. MicroRNA-Mediated Regulation of Vascular Endothelium: From Pro-Inflammation to Atherosclerosis. Int J Mol Sci. 2025;26(13):5919. doi:10.3390/ijms26135919.*
- ***Wang J***, *Li Y, Wang H, Meng Q, Li P, Wang Y, et al. Harnessing miRNA therapeutics: a novel approach to combat heart and brain infarctions in atherosclerosis. Cell Death Discov. 2025;11:482. doi:10.1038/s41420-025-02649-9.*
- ***Letonja J***, *Petrovič D. A Review of MicroRNAs and lncRNAs in Atherosclerosis as Well as Some Major Inflammatory Conditions Affecting Atherosclerosis. Biomedicines. 2024;12(6):1322. doi:10.3390/biomedicines12061322.*
- ***Citrin KM***, *Fernández-Hernando C, Suárez Y. MicroRNA regulation of cholesterol metabolism. Ann N Y Acad Sci. 2021;1495(1):55–77. doi:10.1111/nyas.14566.*

https://doi.org/10.3390/jpm16050236
